# Multiplexed evaluation of immunity against SARS-CoV-2 variants using surface enhanced fluorescence from a nanostructured plasmonic chip

**DOI:** 10.1186/s12951-022-01687-0

**Published:** 2022-12-15

**Authors:** Ruibin Hu, Yang Yang, Ying Liu, Tao Liao, Yiyi Liu, Jiahu Tang, Guanghui Wang, Guoxin Wang, Yongye Liang, Jing Yuan, Bo Zhang

**Affiliations:** 1 Department of Biomedical Engineering, Southern University of Science and Technology of China, Shenzhen, 518055 China; 2 Shenzhen Key Laboratory of Pathogen and Immunity, National Clinical Research Center for Infectious Disease, State Key Discipline of Infectious Disease, Shenzhen Third People’s Hospital, Second Hospital Affiliated to Southern University of Science and Technology, Shenzhen, 518055 China; 3 WWHS Biotech. Inc, Shenzhen, 518055 China; 4 Department of Materials Science and Engineering, Southern University of Science and Technology of China, Shenzhen, 518055 China

**Keywords:** COVID-19, SARS-CoV-2, Acquired immunity evaluation, Neutralizing antibodies, pGOLD, Surface enhanced fluorescence

## Abstract

Generated by the immune system post-infection or through vaccination, the effectiveness of antibodies against emerging SARS-CoV-2 variants is crucial for protecting individuals from the COVID-19 pandemic. Herein, a platform for the multiplexed evaluation of SARS-CoV-2 neutralizing antibodies against various variants was designed on the basis of near-infrared (NIR) surface enhanced fluorescence by nano-plasmonic gold chip (pGOLD). Antibody level across variants (Wild-type, Alpha, Beta, Delta, Omicron) was confirmed by the sera from recovered-individuals who were unvaccinated and had infected with Wild-type, Delta, Omicron variants. However, the neutralizing activity against Omicron variant was markedly decreased for individuals infected by Wild-type (~ 5.6-fold) and Delta variant (~ 19.1-fold). To the opposite, neutralizing antibody from individuals recovered from Omicron variant infection showed weak binding strength against non-Omicron variants. Antibody evolution over time was studied with individuals 196–530 days post Wild-type infection. Decreasing IgG antibody titer accompanied by increasing IgG binding avidity with elongated post-infection period were observed for the sera from Wild-type recovered-individuals with different post-infection times, suggesting that after the primary infection, a great number of antibodies were generated and then gradually decreased, while the antibody matured over time. By comparing the IgG level of individuals vaccinated for 27–51 days with individual post-infection, we found that ca. 1 month after two doses of vaccination, the antibody level was comparable to that of 500 days post-infection, and vaccination could enhance IgG avidity more efficiently. This work demonstrated a platform for the multiplexed, high-throughput and rapid screening of acquired immunity against SARS-CoV-2 variants, providing a new approach for the analysis of vaccine effectiveness, immunity against emerging variants, and related serological study.

**Graphical Abstract:**

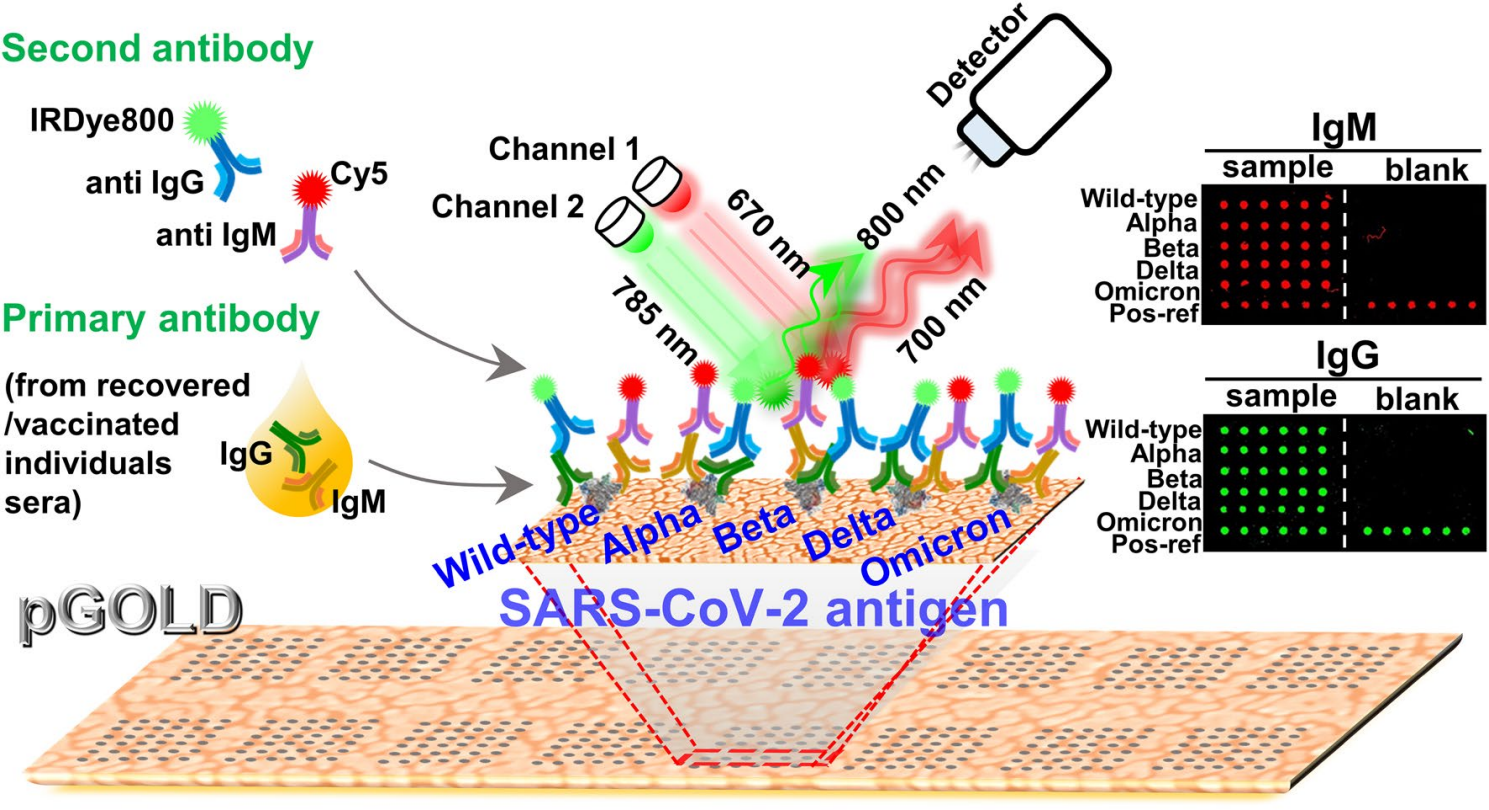

**Supplementary Information:**

The online version contains supplementary material available at 10.1186/s12951-022-01687-0.

## Introduction

Severe acute respiratory syndrome coronavirus 2 (SARS-CoV-2) has caused billions of infections and over six million deaths since its outbreak [1]. With the continuous emergence of variants, the infectivity of the virus has been strengthened, and the number of new cases is still on the rise, threatening public health, economy and social life. Currently, reverse transcription-polymerase chain reaction (RT-PCR) has become the “gold standard” for COVID-19 diagnosis [2, 3]. Although we have overcome the supply shortage issue at disease outbreak, the availability of this method is still limited as it requires relatively complicated equipment and process, not suitable for resource poor regions. Nearly three years since the SARS-CoV-2 outbreak, there has been paradigm shift in the focus of diagnostics: a) from singleplex SARS-CoV-2 test to multiplex test for Wild-type and its variants [4]; b) from solely SARS-CoV-2 diagnosis to acquired immunity assessment (i.e. vaccine effectiveness evaluation) against different SARS-CoV-2 variants [5–8], aiming at reopening of numerous nations. The development of multiplexed antibody neutralization assay platform is thus crucial for the assessment of acquired immune effect over time.

At present, various antibody neutralizing activity tests have been developed using lateral flow assays (LFAs) [9, 10], enzyme linked immunosorbent assay (ELISA) [11, 12] and chemiluminescence (CLIA) techniques [13, 14]. LFA is convenient and rapid, while it is challenging to achieve antibody detection in a sensitive and high-throughput fashion. ELISA and CLIA are classical immunological assays, while they cannot detect multiple biomarkers in a single run. Also, the above methods cannot be applied to evaluate antibody maturation processes [15, 16] and antibody avidity, especially for various variants [17, 18]. Hence, it is urgent to develop a sensitive and high-throughput method to evaluate antibody neutralizing capacity and antibody avidity, to better understand antibody responses against different variants of SARS-CoV-2, antibody maturation processes, and the roles these factors play in the acquired immunity processes.

Stemming from the surface plasmon resonance (SPR) effect, local enhancement of electromagnetic fields near dielectric and metallic surfaces has enabled ultrahigh sensitive sensing for medical applications [19]. Series of nano-structured surface [20] with various fabrication strategy [21], and different modes SPR [22, 23] has been widely explored for biosensing. Recently, there is an increasing trend of applying plasmonic-based biosensors for viral diagnostics [24]. A nano-plasmonic gold chip (pGOLD) was exploited over the past years, and a series of detection modality have been established [25–28]. The chip is composed of nanoscale gold islands with abundant nano-gaps which is capable of enhancing near-infrared (NIR) fluorescence owing to plasmonic resonance and local electric field enhancements effect [26]. The highly sensitive fluorescence enhancement effect on the pGOLD can be used for multiplexed biomarkers analysis with 6 orders of dynamic range [25]. However, the pGOLD platform has not been investigated for screening of acquired immunity against multiple virus and its variants.

To meet the needs for evaluation of acquired immunity against different SARS-CoV-2 variants, in this study, based on the pGOLD, we have constructed a platform that can simultaneously evaluate the neutralization effectiveness of SARS-CoV-2 specific antibodies against different variants with high sensitivity and high-throughput. Analysis of sera samples from individuals who recovered from infection with different variants revealed that SARS-CoV-2 specific antibodies had different degrees of cross-neutralizing capacity against Wild-type and Alpha, Beta, Delta, Omicron variants. Consistent with the immune escape discovered for Omicron variant [29, 30], the neutralization effectiveness against Omicron variant is sharply reduced for Wild-type and Delta variant infected individuals, and neutralization effectiveness for Omicron infected individuals was also weaker against non-Omicron variants. Furthermore, the sera from the Wild-type infected individuals with different post-infection times and the individuals who completed two doses of vaccine were collected to investigate IgG antibody evolution over time. We found that both infected and vaccinated individuals had different degrees of acquired immunity against major SARS-CoV-2 variants. The overall IgG level decreased over time, on the contrary, the antibody avidity increased, suggesting the antibodies was trend to mature over time. Taken together, the pGOLD based highly-sensitive assay platform can realize high dilution of clinical samples to avoid the non-specific signals, which is of great significance in the effective evaluation of SARS-CoV-2 antibodies, and the assay can be easily expanded to emerging variants.

## Materials and methods

### Materials

pGOLD chips were purchased from Nirmidas Biotech Inc. (Palo Alto, USA). The glass chips coated by amino silane were purchased from Corning incorporated (New York, USA). Tween-20, PBS and BSA were purchased from Sigma-Aldrich (Shanghai, China). IRDye800-NHS ester and Cy5-NHS ester were purchased from Licor Biosciences (Nebraska, USA). Fetal bovine serum (FBS) was purchased from Invitrogen (Carlsbad, USA). Recombinant human IgG and human IgM were purchased from Acro biosystems. Goat anti-human IgG antibody and goat anti-human IgM were purchased from Sino Biological. The Wild-type S1 (cat. 40,591-V08H3), Alpha variant S1 (cat. 40,591-V08H12), Beta variant S1 (cat. 40,591-V08H15), Delta variant S1 (cat. 40,591-V08H23) and Omicron variant S1 (cat. 40,591-V08H41) were purchased from Sino Biological. The sera of various variants recovered-individuals were provided by Affiliated Hospital of South University of Science and Technology. And 23 control samples (collected before the SARS-CoV-2 outbreak) were acquired from the WWHS Biotech. Inc. (Shenzhen, China). Other chemicals used in this study were of analytic grade. Ultrapure water (≥ 18.2 MΩ/cm), purified using a Milli-Q purification system (Millipore), was used in all experiments.

### The secondary antibody labeled with fluorophores

Briefly, IRDye800-NHS ester was dissolved in dry dimethyl sulfoxide (DMSO), to prepare a 2 mM solution. Then, 90 µL 11 µM goat anti-human IgG was carried out in phosphate buffered saline (PBS) solution. It had been mixed well and reacted at room temperature for one hour when 10 µL IRDye800 solution was added into anti-human IgG solution. Meanwhile, the separation column NAP-5 (GE-Healthcare, USA) was washed several times before use. The reaction mixture was loaded into the column, and then it was added with 400 µL PBS in it. Finally, collect the solution coming out after adding 500 µL PBS, resulting in a solution of 2 µM anti-human IgG-IRDye800. Store the solution in 4 °C for later use. The anti-human IgM-Cy5 was labeled as the above protocol.

### SARS-CoV-2 antigen microarray printing

Each pGOLD chip was printed with five SARS-CoV-2 antigens including Wild-type, Alpha, Beta, Delta, and Omicron epitope of the spike protein (S1) by a microarray printing robot (GeSiM Nano-Plotter 2.1, Germany) at the concentrations of 50 µg mL^−1^. 36 spots with 6 row and 6 column were printed in every well and the spot features diameter of ca. 400 μm. On each well, the mixture of 10 µg mL^−1^ recombinant human IgG and 100 µg mL^−1^ recombinant IgM were also printed to serve as a positive internal reference and signal normalization. Identical microarrays were printed on 16 isolated wells in each pGOLD chip with a total of 50 chips. When in use, each plate was incubated with the FAST frame incubation chamber (Milliporea Sigma). The antigen-printed chips were vacuum-sealed and stored at −80 °C for later use.

### Microarray sandwich assay procedure

Fit the antigen-printed chip in FAST frame incubation chamber. Added then 100 μL 50 mg mL^−1^ BSA in PBS solution into each well when the chip was dried and incubated at room temperature for one hour to block the sites not occupied by antigen. The well should be kept wet, otherwise, the non-specific signal and the background signal would be high. Tween-20 helped prevent the well from completely drying. Each well was then sucked the BSA blocking solution out and washed with PBST solution (PBS solution plus 0.05% Tween-20) one time. And 100 µL of 100 × diluted patient serum in FBS was then added into each well and incubated at room temperature for one hour. A positive control (diluted serum inoculated with COVID-19 vaccine) and blank control (FBS only) were also included in each chip. And the mixture of recombinant human IgG and IgM on each well for intrawell signal normalization to minimize the effect of slight differences in pGOLD uniformity across each chip. Each well was subsequently incubated with a mixture of 2 nM (diluted in BSA solution) IRDye800-labelled anti-human IgG secondary antibody, 2 nM (diluted in BSA solution) Cy5-labelled anti-human IgM secondary antibody for 20 min at room temperature, with gentle shaking. Chips were sucked out and washed five times with PBST (last time 15 min) and three times with PBS (last time 5 min). Take the chip from the incubation chamber, put it into a container with deionized water, and shake it gently at room temperature for 1 min. Then subsequent dry it with dryer.

### Fluorescence measurement and signal analysis

After the incubation procedures finished, a dual-channel MidaScan microarray scanner (Nirmidas Biotech) was used to scan each chip for IRDye800-labeled anti-human IgG and Cy5-labeled anti-human IgM signals with 785 nm channel and 670 nm channel, respectively. The mean fluorescence intensity (MFI) signal for each spot was quantified by MidaScan Software version 2.0.0. And the MFI of each spot was analyzed by background signal subtracted, and removed the highest and lowest MFI of the antigen spots for each channel, which was used to analyze antibody level and antibody avidity in each sample. Subsequently, the MFI was divided by the average positive control MFI signal, resulting in an intrawell ratio for IgG and IgM. The antibody level was used to determine the antibody status of the samples for the corresponding various antigens. The cutoff was calculated by blank fluorescent signal plus three standard deviations. All the data were analyzed by the origin 2017 pro.

## Results and discussion

### The principle of COVID-19 acquired immunity evaluation platform based on pGOLD

**Fig. 1:**
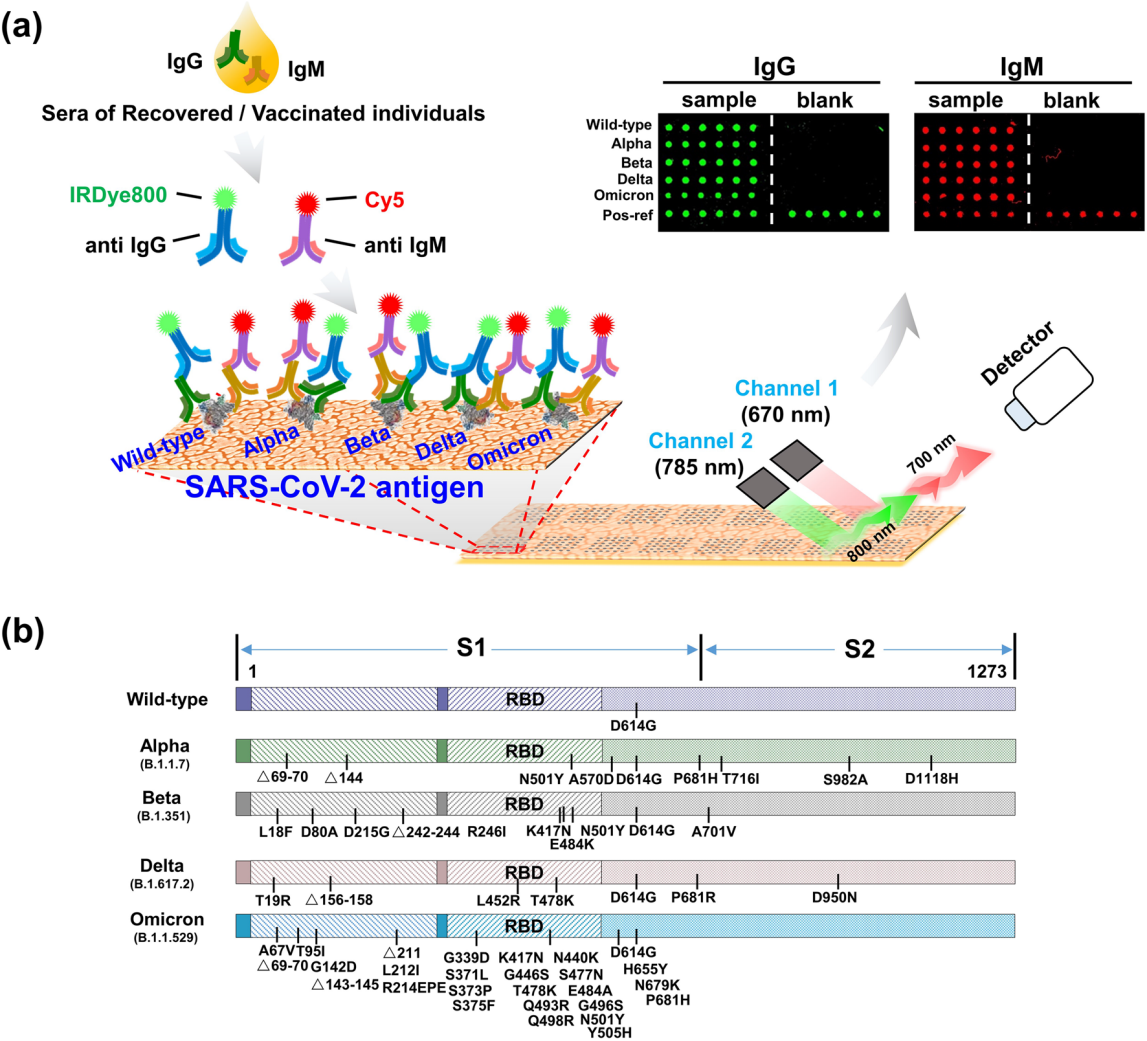
Scheme of SARS-CoV-2 variants array on pGOLD for IgG/IgM profiling. **a** Wild-type, Alpha, Beta, Delta, Omicron variants of epitope of the spike protein (S1) as well as a mixture of recombinant human IgG and IgM as the positive reference were arrayed on the pGOLD substrate. The secondary antibodies labeled with IRDye800 and Cy5 were bound to the primary antibody IgG, IgM after they were specifically bound to the antigens. The chips were imaged by the scanner with 785 nm channel for IgG detection and 670 nm channel for IgM detection. The positive reference in each block served as inter-block signal normalization. **b** The detailed information of S1 mutation sites of different variants in this study

Each block on the pGOLD was arrayed with SARS-CoV-2 Wild-type epitope of the spike protein (S1), and its Alpha, Beta, Delta, Omicron variants, together with a positive reference (Pos-ref, mixture of recombinant human IgG and IgM) (Fig. 1a). Followed by blocking and incubation with sera from post-infection or vaccinated individuals, the immobilized antigens will capture human IgG and IgM antibodies specific to them. The captured human IgG and IgM antibodies were then labeled with IRDye800 conjugated anti-human IgG and Cy5 conjugated anti-human IgM secondary antibody, respectively. The level of IgG and IgM antibodies bound to each antigen spot was reflected by the fluorescent intensity quantified by a confocal microscopy scanner in 670 nm and 785 nm channels (Fig. 1a). Figure 1b shows the detailed information of S1 mutation sites for different variants of the SARS-CoV-2 used in this study.

### Characterization and sensitivity of the platform for SARS-CoV-2 neutralizing antibody assays

**Fig. 2:**
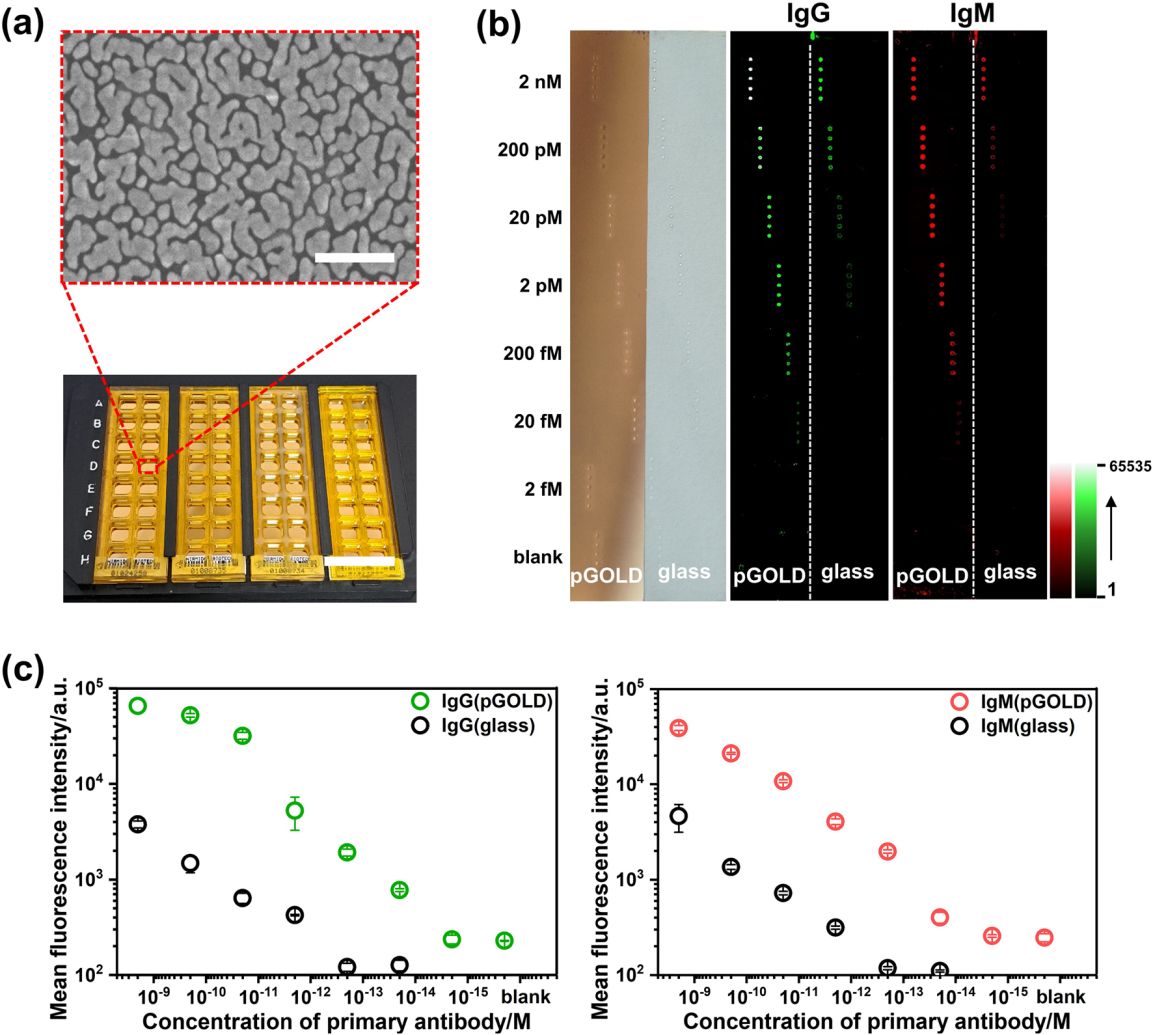
Characteristics of pGOLD and titration curve for SARS-CoV-2 antibody detection. **a** The SEM image (up) of pGOLD substrate before arraying the antigens of SARS-CoV-2, the scale bar is 500 nm; the photograph of a set of pGOLD (down), the scale bar is 2 cm. **b** Recombinant human IgG and IgM antibodies to Wild-type S1 of SARS-CoV-2 with known concentration were serial diluted from 2 nM to 2 fM in fetal bovine serum (FBS), then assayed on pGOLD and glass substrates. As shown in **b**, the left slide is the pGOLD substrate, and the right part is glass substrate. On pGOLD, the fluorescent signal was saturated at 2 nM of IgG, with limit of detection down to 20 fM, over two orders of magnitudes lower than on glass substrate. **c** The titration curves and dynamic range of SARS-CoV-2 specific IgG and IgM antibody detection on pGOLD and glass substrates. The mean fluorescence intensity (MFI) was calculated from five array spots for each concentration. The error bars represented the standard deviation of the MFI over the three replicate measurements

Scanning electron microscopy (SEM) image showed the nano-structured plasmonic substrate was consist of dense nanoscale gold islands with abundant nano-gaps (Fig. 2a). The sensitivity of the proposed assay on the basis of pGOLD was then investigated by recombinant human IgG and IgM antibodies against Wild-type SARS-CoV-2 S1. The limit of detection (LoD) was down to 20 fM, boosting the fluorescent signal by at least 100 folds on pGOLD than on a glass (Fig. 2b, c). With over five orders of magnitude dilution of recombinant human SARS-CoV-2 IgG and IgM antibodies, the corresponding signal error bar value for statistical mean fluorescence intensity (MFI) was still small, demonstrating high reproducibility and consistency of the proposed pGOLD platform (Fig. 2c). The results suggested high sensitivity antibody detection and wide dynamic range of the pGOLD assay for variant antigens of SARS-CoV-2. This advantage allows clinical samples to be highly diluted to avoid the generation of nonspecific signals from complex biological matrices, which is conducive to the accurate evaluation of the neutralization of SARS-CoV-2 specific antibodies against different variants.

### Performance of the pGOLD platform in antibody detection for clinical samples

**Fig. 3:**
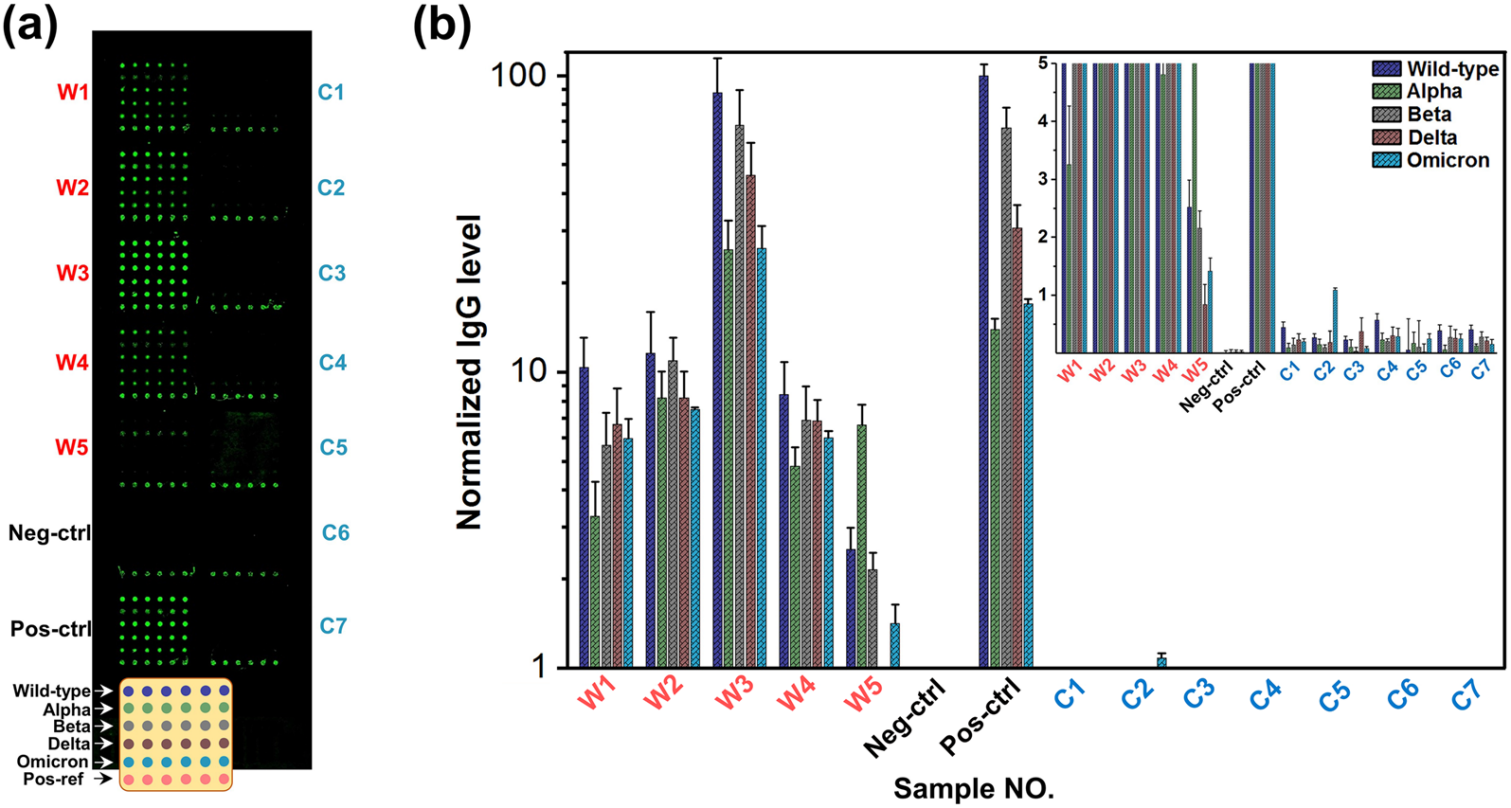
Detection of SARS-CoV-2 specific IgG antibody in clinical samples. **a** A confocal fluorescence image of IgG signals in Wild-type infected individual sera (n = 5, denoted W1-W5) and negative samples (n = 7, denoted C1-C7), sera from vaccinated volunteer as positive control (denoted Pos-ctrl) and fetal bovine serum (FBS) as negative control (denoted Neg-ctrl); 100 µg per mL recombinant human IgG was used as a positive internal reference (denoted Pos-ref) for normalization of fluorescent signals. All sera were diluted 100 times in FBS. **b** Mean fluorescence intensity (MFI) of IgG against various antigens measured from pGOLD seen in (a), with background signals subtracted. Error bars indicate standard deviation from the MFI signals detected against the respective microarrayed antigen spots (n = 6)

After establishing the highly sensitive antibody detecting platform based on pGOLD, we next focused on investigating the performance of our platform in detection of SARS-CoV-2 neutralizing antibody in clinical sera. The conditions of serum dilution to improve the signal-to-noise ratio (SNR) was optimized firstly. With a 100 folds dilution of serum in fetal bovine serum (FBS), the background signal was close to intact pGOLD substrate, leading to optimal SNR (Additional file 1: Fig. S1). Notably, although without the introduction of fluorescent molecule, the chip still has a certain background signal, which may be related to the materials of glass substrate and the nano-gold film on the surface. But it does not affect the experimental results. Subsequently, sera of individuals recovered from SARS-CoV-2 ancestral virus infection and individuals before COVID-19 outbreak were applied as positive and negative samples to demonstrate the clinical validity of the platform. For Wild-type recovered-individual sera, we observed positive signals in different degree for IgG antibody against all variants arrayed on pGOLD substrate, including Wild-type, Alpha, Beta, Delta, and Omicron; and there was barely any fluorescent signal for negative controls (Fig. 3a). To improve the accuracy of analysis, recombinant human IgG was introduced into each block as a positive internal reference. Based on this, we normalized the fluorescence signal in Fig. 3a and obtained the fluorescence intensity value (Fig. 3b, Additional file 1: Table S1). The fluorescence signal intensity of each variant S1 was intuitively displayed. The results not only proved the reliability of the proposed method, but also showed that the recovered individuals had produced specific antibodies against SARS-CoV-2, and these antibodies had varying degrees of neutralizing effect on variants S1 due to individual differences.

### Assessments of cross-neutralizing effectiveness

**Fig. 4:**
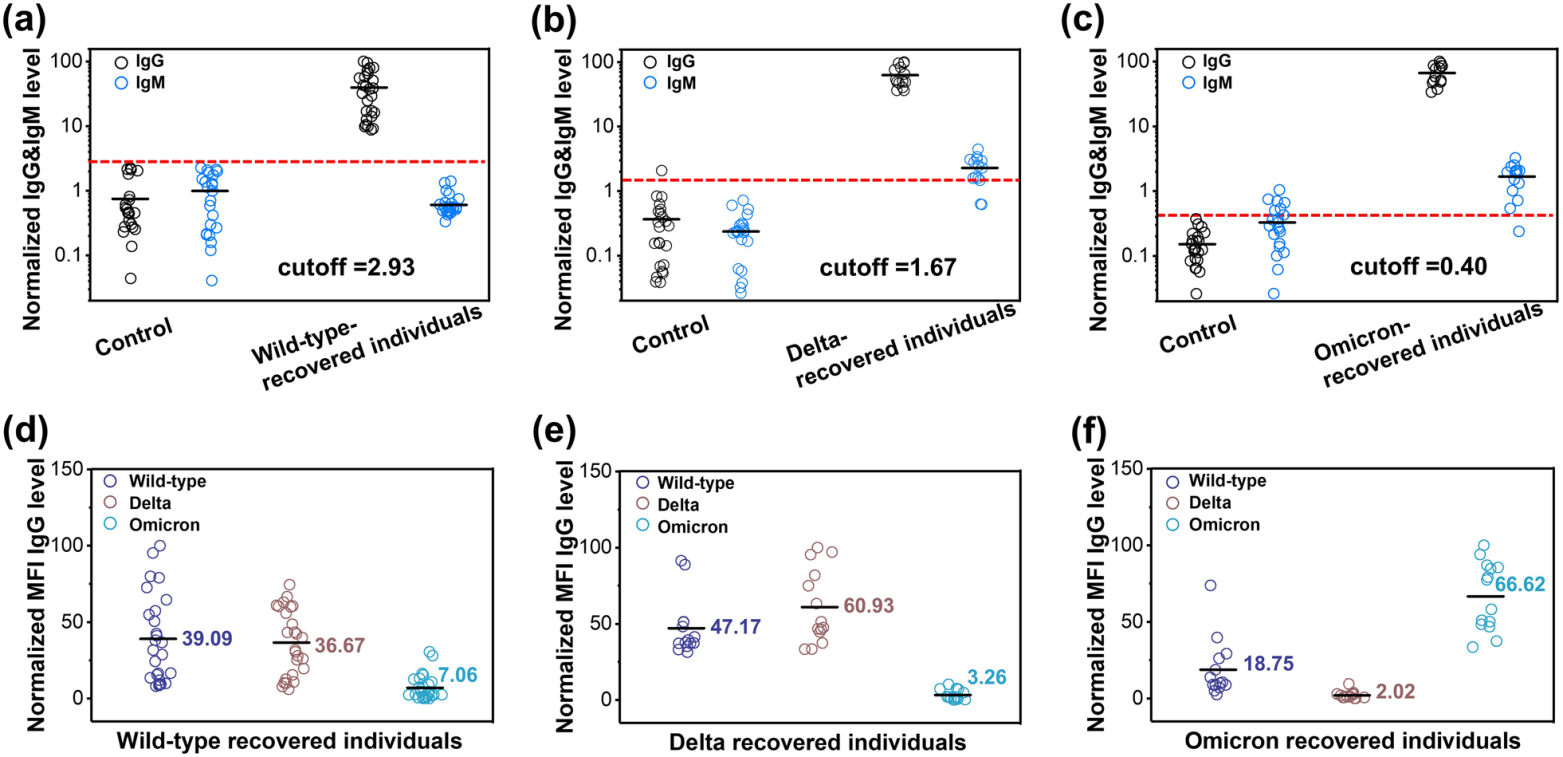
Multiplexed antibody neutralizing test against various variants for post-infection individuals. **a** Neutralizing effectiveness of IgG and IgM were evaluated by Wild-type recovered-individual sera against Wild-type S1. **b** Neutralizing effectiveness of IgG and IgM were evaluated by Delta recovered-individual sera against Delta S1. **c** Neutralizing effectiveness of IgG and IgM were evaluated by Omicron recovered-individual sera against Omicron S1. **d** The cross-neutralizing capacity of Wild-type recovered-individual sera. **e** The cross-neutralizing capacity of Delta recovered individual sera. **f** The cross-neutralizing capacity of Omicron recovered-individual sera. Assessments of cross-neutralizing effect was calculated by normalized mean fluorescence intensity (MFI) of IgG and IgM detected in sera from post-infection individuals. The red dash line indicates the normalized MFI of IgG of control group plus triples of standard deviation. The black line indicates the mean of normalized MFI of IgG. (Control: sera collected before the COVID-19 outbreak (n = 23); Wild-type recovered-individual sera (n = 26); Delta recovered-individual sera (n = 14); Omicron recovered-individual sera (n = 14)

Next, 26 Wild-type recovered-individual sera, 14 Delta recovered-individual sera, 14 Omicron recovered-individual sera (confirmed by sequencing) were introduced to investigate the neutralizing effectiveness of SARS-CoV-2 specific antibodies against those variants. At the same time, 23 serum samples not infected with COVID-19 were used as negative controls. The results showed that the IgG level in the clinical sera was significantly higher than that in the control group, while the IgM level was almost the same (Fig. 4a–c, Additional file 1: Table S2a–c), since IgM antibody was produced early in an immune response and gradually faded, to be replaced by IgG antibody [31, 32]. High level of IgM indicated an acute infection [33, 34]. In our study, there was no significant difference of IgM level between the control group and the recovered patients, meaning most of IgM had been converted into IgG, and the LoD had been below the threshold cutoff.

Subsequently, the cross-neutralizing capacity of SARS-CoV-2 antibodies was evaluated. The results showed that sera from Wild-type recovered-individuals efficiently neutralized against Wild-type and Delta variant with a higher efficacy but showed over 5 folds reduced neutralization against Omicron variant (Fig. 4d, Additional file 1: Table S3a). Similarly, sera from Delta variant recovered-individuals efficiently neutralized against Delta variant and Wild-type with a higher efficacy but showed over 19 folds reduced neutralization against Omicron variant (Fig. 4e, Additional file 1: Table S3b). For sera from Omicron recovered-individuals, the result was reversed, they exhibited strong immunity against Omicron variant but with minimal neutralization potency against Wild-type and Delta variant (Fig. 4f, Additional file 1: Table S3c). The above results indicated that Omicron variant had immune escape, especially for sera from non-Omicron-infected recovered individuals. This was consistent with prior reports [29, 35–37], although the limitations of this study include the limited serum samples, and the use of IgG targeting subunit spike protein in vitro may not reflect the real situation of virus particle targeting antibody in vivo.

### Antibody level and avidity in recovered and vaccinated individuals

To investigate the antibody development process for vaccinated individuals, a multiplexed antibody level and avidity assay was developed for SARS-CoV-2 Wild-type and its variants on our pGOLD platform. Through urea treatment after sera incubation to remove weakly bound antibodies, the ratio between IgG signals with/without urea treatment is represented as avidity [28]. Based on this, the sera of a vaccinated volunteer were collected at different time points after SARS-CoV-2 vaccination to study antibody level and avidity. The results indicated that IgG against various variants showed relatively low level and avidity before the second dose of vaccine (Additional file 1: Fig. S2, S3 and Table S4). The second dose of vaccine significantly increased IgG level and boost IgG avidity against all SARS-CoV-2 variants (measured a week after the second dose). With the extension of time post vaccination (7 weeks after second dose), the IgG level and avidity further increased (Additional file 1: Fig. S2, S3 and Table S4). These results demonstrated that the IgG could increase over time after vaccination, and a second dose of vaccine could efficiently boost IgG avidity (the “maturation” process).

**Fig. 5:**
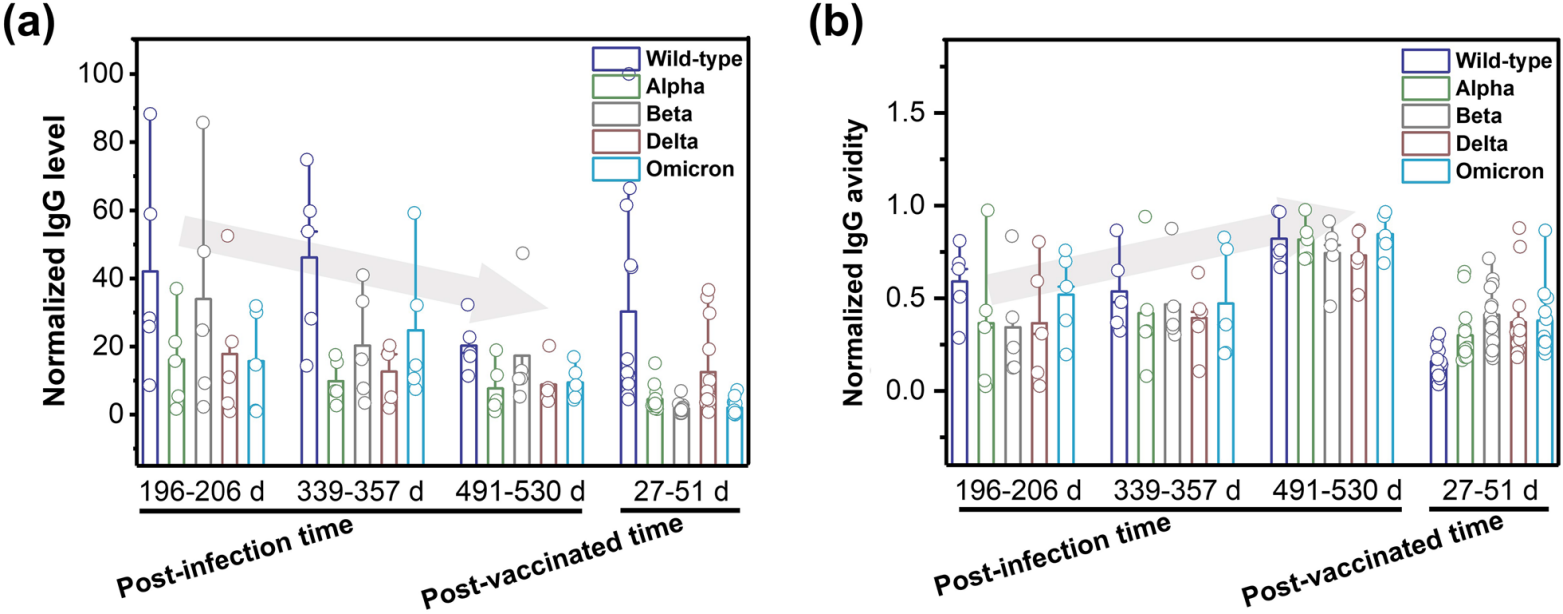
IgG level and avidity over time for post-infection and vaccinated individuals. **a** Normalized IgG level against SARS-CoV-2 various variants at different time points in post-infection and vaccinated individuals; **b** Normalized IgG avidity against various SARS-CoV-2 variants at different time points in post-infection and vaccinated individuals. Columns showed mean level and avidity of IgG according to the days between symptom onset to sample collection or between the second dose of vaccine to sample collection. A colorful circle on each column represents a clinical sample

Subsequently, the sera from Wild-type recovered-individuals (without COVID-19 vaccination) within 196–206, 339–357, 491–530 days post-infection, as well as the sera of individuals who completed two doses of vaccine within 27–51 days were grouped to investigate the IgG antibody evolvement over time. We observed a decreasing trend of IgG level with the increase of post-infection time (196–530 days, Fig. 5a and Additional file 1: Table S5a) in spite of the variation between individuals, matching the nature of IgG life-cycle [38]. The IgG level of the individuals who completed two doses of vaccine within 27–51 days were comparable to individuals ca.500 days post-infection (Fig. 5a and Additional file 1: Table S5b), implying that the antibody level was largely increased after a period of vaccination. Despite the variation between individuals, with the extension of time (from ca.200 days, ca.350 days to ca.500 days), an increase of IgG avidity was observed (Fig. 5b and Additional file 1: Table S3). In particular, the value of IgG avidity was largely over 0.5 against all variants after 500 days of exposure, suggesting the evolving of highly specific antibody over time [39]. Also, the avidity of IgG generated by vaccination matched those ca.350 days post-infection (Fig. 5b and Additional file 1: Table S5) in a relatively shorter period of time (27–51 days). Although the samples are limited, the results may reflect that multi-dose of vaccine can efficiently increase antibody level and have a broadened neutralizing capacity against major variants of concern, so as to protect the individuals from the COVID-19 pandemic.

## Conclusions

Timely assessment of the effectiveness of acquired immunity against previous and emerging variants with high sensitivity and high-throughput is an unprecedented need introduced by the COVID-19 pandemic. Utilizing the unique nanostructure characteristics of pGOLD, we have developed a platform for evaluating the neutralizing effect of COVID-19 specific antibodies against different variants in a high-throughput and multiplexed format. Recombinant human IgG and IgM were introduced to validate the sensitivity of the proposed approach. The sensitivity of 20 fM was achieved, at least two orders of magnitude improvement over assay on the glass substrate. This sensitive detection method can allow clinical samples to be highly diluted, so as to avoid the deviation of experimental results caused by non-specific signals which brought by non-target biomolecules from complex biological matrix. The sera of Wild-type recovered-individuals and controls (before the COVID-19 outbreak) were collected to validate the multiplexed assay on this platform. The result indicated that the IgG level of recovered group was significantly higher than control group, demonstrating the capacity of pGOLD to detect antibodies against various variants. Meanwhile, it suggested that a large number of SARS-CoV-2 specific antibodies have been not only generated in recovered individuals, they also specifically bound to various variants.

Cross-neutralizing test showed that Wild-type recovered-individual sera effectively neutralizes Wild-type and Delta variant with a high level, but the neutralizing effect for Omicron variant decreased sharply. There was a similar phenomenon in Delta recovered-individual sera. However, the neutralizing effect of Omicron recovered-individual sera on Omicron variants was significantly stronger than that on Wild-type and Delta variants. It can be explained that Omicron variants almost cover the key mutations of Alpha, Beta & Delta variants, including K417N, E484A, and N501Y and other known mutations which are proved to alter the susceptibility of the virus to neutralization by protective antibodies [40–43]. Furthermore, the sera from the Wild-type recovered-individuals within different post-infection times were collected to investigate IgG development over time, and it was found that with the extension of time, the level of IgG gradually decreased. On the contrary, the overall IgG avidity showed an increased trend, in spite of variation between individuals, suggesting that the antibody was matured over time, although their amount was decreased if no secondary infection was introduced. Similar results were found in the sera of volunteer vaccinated with different doses of vaccine. Also, it was observed that the IgG level of individuals who had the second dose of vaccine within 27–51 days was equivalent to that ca. 500 days after infection and their antibody has a broadened neutralizing activity against major variants of concern, meaning multi-doses vaccine can facilitate the ending of COVID-19 pandemic.

Overall, multiplexed SARS-CoV-2 antibody neutralizing assessment platform was able to profile antibody response against various variants, shedding light on antibody level and avidity for post-infection and vaccinated individuals. With the scalability nature of this approach, new variant could be readily integrated onto the chip, which is convenient for us to study the cross-neutralizing effect of antibodies against emerging variants. We believe that the platform can provide a feasible solution for long-term tracking of immune response, drug development and prevention of viral pandemic in the future.

## Supplementary Information

**Additional file 1: Fig. S1.** Optimization of serum incubation condition. **(a)** Confocal fluorescence scanning images and **(b)** Mean fluorescent intensity (MFI) background signal, compared to whole serum incubation or 1:1 dilution in FBS, 1:10 dilution in FBS, 1:100 dilution in FBS improves signal background ratio. **Fig. S2.** Confocal fluorescence scanning images of IgG acquired after testing serum samples in isolated wells. The right part of image showed the amount of antibody at different times of vaccination. The left part of image showed the antibody avidity against various variants after urea treated at different times of vaccination. **Fig. S3.** Vaccination induced antibody and IgG avidity against SARS-CoV-2 variants of concern. (a) The level of IgG was from a vaccinated individual, at different time point. The data was normalized by the IgG mean fluorescent intensity (MFI) signal. (b)The avidity of IgG produced by a vaccinated individual, at different time point. Antibody avidity was calculated by the MFI of the urea-treated samples divided by the MFI of the non-treated samples. The sera were collected from a volunteer who injected vaccine of SARS-CoV-2 the day before the second dose (1#), one week after the second dose (2#), and seven weeks after the second dose (3#), respectively. **Table S1.** Dataset of SARS-CoV-2 IgG level of Wild-type recovered-individuals and control (before COVID-19 outbreak). **Table S2a**. Dataset of SARS-CoV-2 IgG and IgM level in sera of recovered-individuals who infected with ancestral virus (Wild-type). **Table S2b.** Dataset of SARS-CoV-2 IgG and IgM level in sera of recovered-individuals who infected with Delta variant. **Table S2c.** Dataset of SARS-CoV-2 IgG and IgM level in sera of recovered-individuals who infected with Omicron variant. **Table S3a.** Dataset of IgG MFI of Wild-type recovered-individual sera against variants of concern. **Table S3b.** Dataset of IgG MFI of Delta recovered-individual sera against variants of concern. **Table S3c.** Dataset of IgG MFI of Omicron recovered-individual sera against variants of concern. **Table S4.** Dataset of IgG level and avidity of a volunteer who injected COVID-19 vaccine at different time. **Table S5a.** Dataset of IgG level and avidity of individuals who recovered from Wild-type infection. **Table S5b.** Dataset of IgG level and avidity of volunteers who completed two doses of COVID-19 vaccine.

## Data Availability

The data are all available upon request.
