# Supplementary material for: Multiplexed evaluation of immunity against SARS-CoV-2 variants using surface enhanced fluorescence from a nanostructured plasmonic chip
Source: J Nanobiotechnology. 2022 Dec 15;20:533. doi: 10.1186/s12951-022-01687-0 (PMC9753017; doi:10.1186/s12951-022-01687-0)
Supplement: Supplementary file 1 — Additional file 1: Fig. S1. Optimization of serum incubation condition. (a) Confocal fluorescence scanning images and (b) Mean fluorescent intensity (MFI) background signal, compared to whole serum incubation or 1:1 dilution in FBS, 1:10 dilution in FBS, 1:100 dilution in FBS improves signal background ratio. Fig. S2. Confocal fluorescence scanning images of IgG acquired after testing serum samples in isolated wells. The right part of image showed the amount of antibody at different times of vaccination. The left part of image showed the antibody avidity against various variants after urea treated at different times of vaccination. Fig. S3. Vaccination induced antibody and IgG avidity against SARS-CoV-2 variants of concern. (a) The level of IgG was from a vaccinated individual, at different time point. The data was normalized by the IgG mean fluorescent intensity (MFI) signal. (b)The avidity of IgG produced by a vaccinated individual, at different time point. Antibody avidity was calculated by the MFI of the urea-treated samples divided by the MFI of the non-treated samples. The sera were collected from a volunteer who injected vaccine of SARS-CoV-2 the day before the second dose (1#), one week after the second dose (2#), and seven weeks after the second dose (3#), respectively. Table S1. Dataset of SARS-CoV-2 IgG level of Wild-type recovered-individuals and control (before COVID-19 outbreak). Table S2a. Dataset of SARS-CoV-2 IgG and IgM level in sera of recovered-individuals who infected with ancestral virus (Wild-type). Table S2b. Dataset of SARS-CoV-2 IgG and IgM level in sera of recovered-individuals who infected with Delta variant. Table S2c. Dataset of SARS-CoV-2 IgG and IgM level in sera of recovered-individuals who infected with Omicron variant. Table S3a. Dataset of IgG MFI of Wild-type recovered-individual sera against variants of concern. Table S3b. Dataset of IgG MFI of Delta recovered-individual sera against variants of concern. Table S3c. Dataset of Ig [file 12951_2022_1687_MOESM1_ESM.docx]

**Multiplexed evaluation of immunity against SARS-CoV-2 variants using surface enhanced fluorescence from a nanostructured plasmonic chip**

Ruibin Hu^1,^ **^†^**, Yang Yang^2,^ **^†^**, Ying Liu^1^, Tao Liao^3^, Yiyi Liu^1^, Jiahu Tang^1^, Guanghui Wang^1^, Guoxin Wang^3^, Yongye Liang^4,^*, Jing Yuan^2,^*, Bo Zhang^1,^*

^1^Department of Biomedical Engineering, Southern University of Science and Technology of China, Shenzhen 518055, China;

^2^Shenzhen Key Laboratory of Pathogen and Immunity, National Clinical Research Center for infectious disease, State Key Discipline of Infectious Disease, Shenzhen Third People’s Hospital, Second Hospital Affiliated to Southern University of Science and Technology, Shenzhen518055, China;

^3^WWHS Biotech. Inc., Shenzhen518055, China;

^4^Department of Materials Science and Engineering, Southern University of Science and Technology of China, Shenzhen 518055, China;

***Correspondence:** zhangb3@sustech.edu.cn; yuanj2020@mail.sustech.edu.cn; liangyy@sustech.edu.cn

† These authors contributed equally to this work.

**
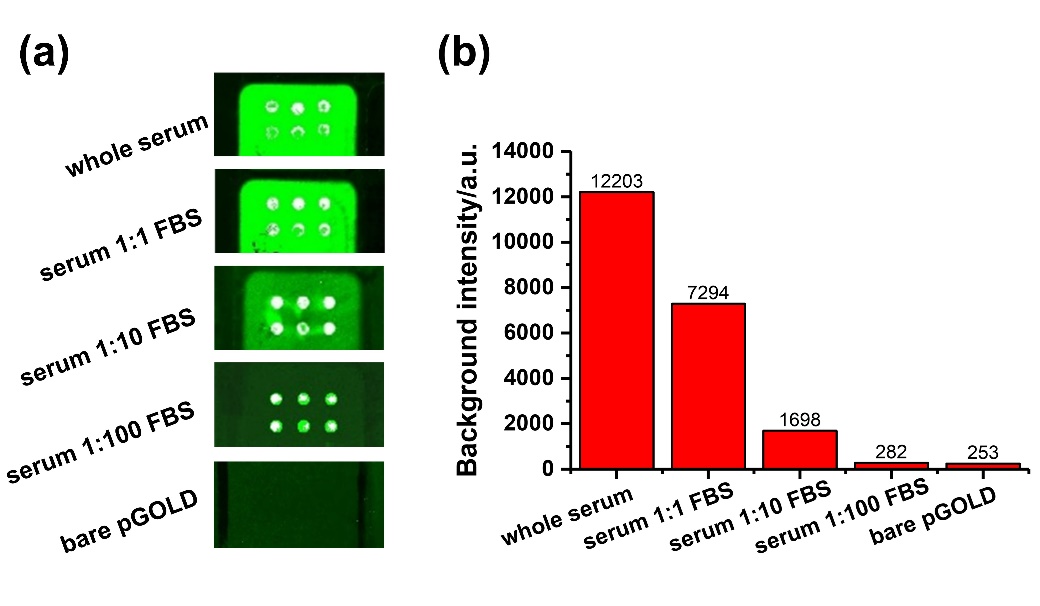
**

**Fig. S1** Optimization of serum incubation condition. **(a)** Confocal fluorescence scanning images and **(b)** Mean fluorescent intensity (MFI) background signal, compared to whole serum incubation or 1:1 dilution in FBS, 1:10 dilution in FBS, 1:100 dilution in FBS improves signal background ratio.


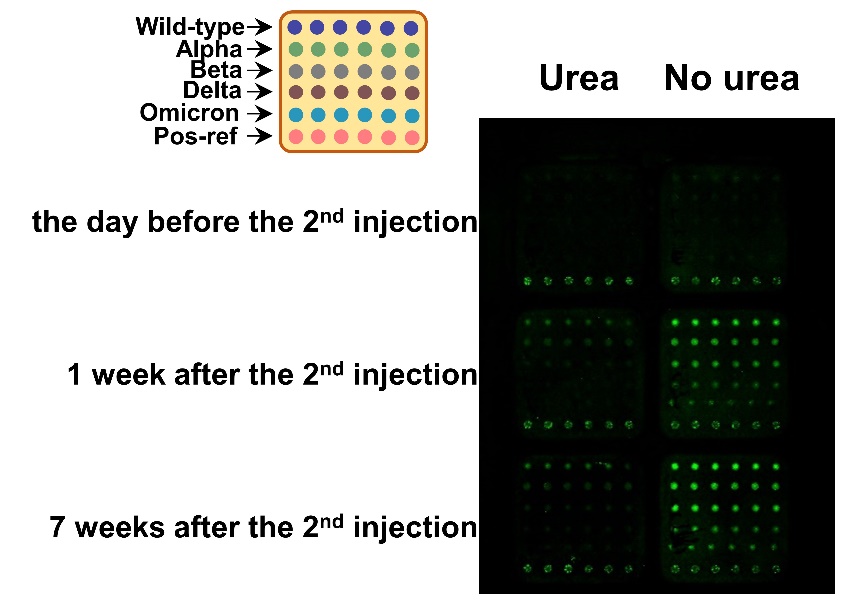


**Fig. S2** Confocal fluorescence scanning images of IgG acquired after testing serum samples in isolated wells. The right part of image showed the amount of antibody at different times of vaccination. The left part of image showed the antibody avidity against various variants after urea treated at different times of vaccination.


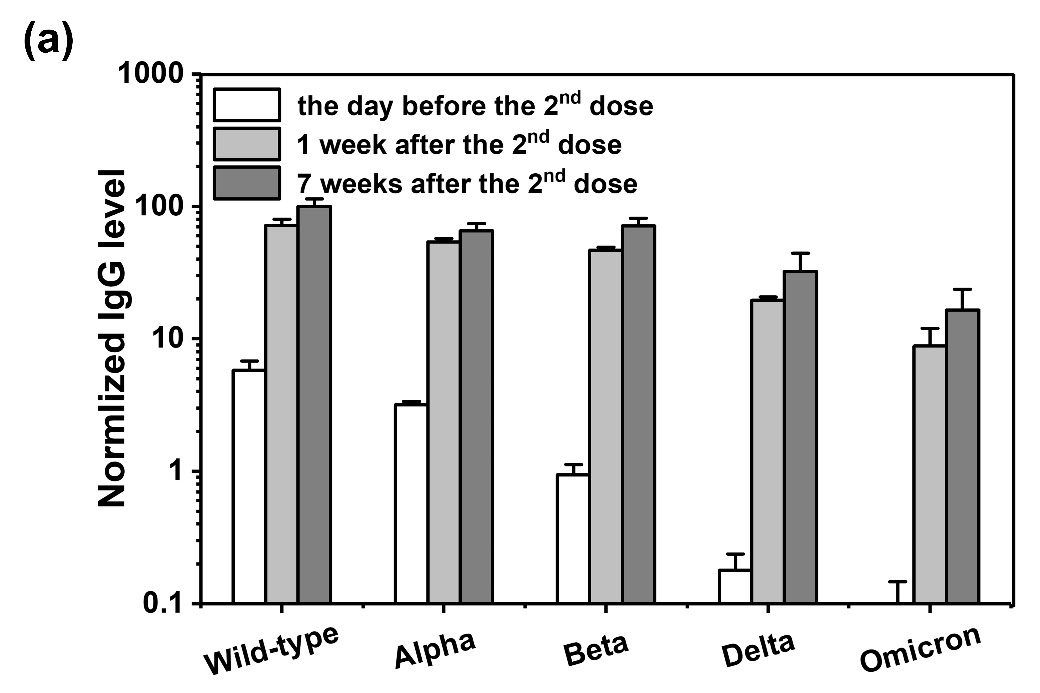


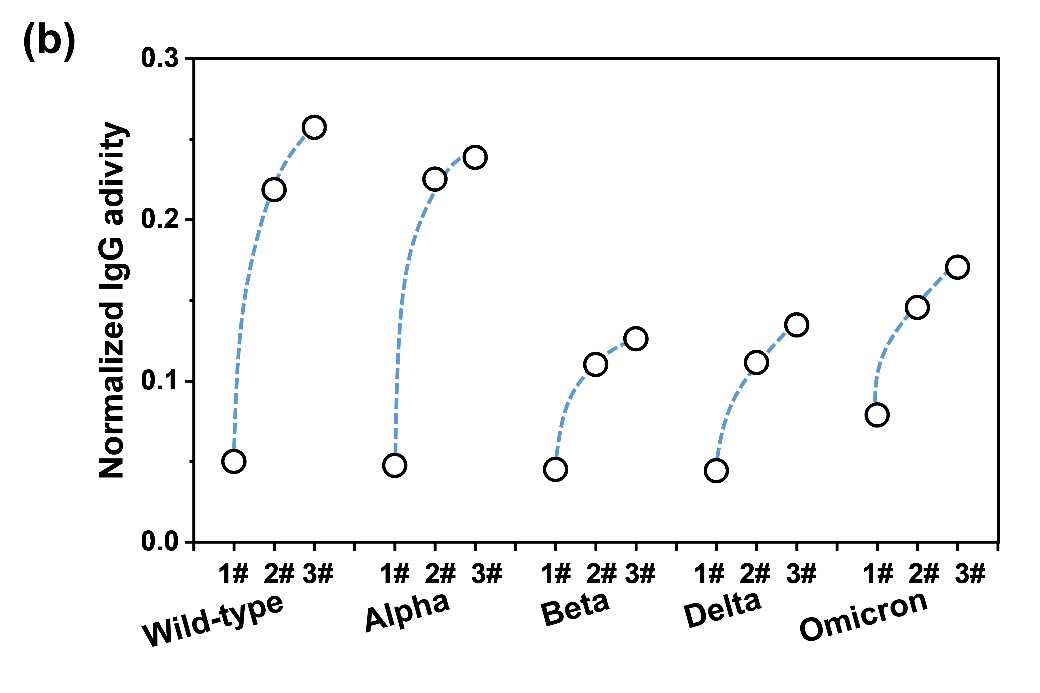


**Fig. S3** Vaccination induced antibody and IgG avidity against SARS-CoV-2 variants of concern. (a) The level of IgG was from a vaccinated individual, at different time point. The data was normalized by the IgG mean fluorescent intensity (MFI) signal. (b)The avidity of IgG produced by a vaccinated individual, at different time point. Antibody avidity was calculated by the MFI of the urea-treated samples divided by the MFI of the non-treated samples. The sera were collected from a volunteer who injected vaccine of SARS-CoV-2 the day before the second dose (1#), one week after the second dose (2#), and seven weeks after the second dose (3#), respectively.

**Table S1** Dataset of SARS-CoV-2 IgG level of Wild-type recovered-individuals and control (before COVID-19 outbreak)

| Sample ID | Antigen | MFI | Sample ID | Antigen | MFI |
| --- | --- | --- | --- | --- | --- |
| W1 | Wild-type S1 | 3837.02 | C1 | Wild-type S1 | 170.47 |
|  | Alpha S1 | 1213.18 |  | Alpha S1 | 40.82 |
|  | Beta S1 | 2100.21 |  | Beta S1 | 58.61 |
|  | Delta S1 | 2478.86 |  | Delta S1 | 90.82 |
|  | Omicron S1 | 2219.01 |  | Omicron S1 | 78.73 |
| W2 | Wild-type S1 | 4310.65 | C2 | Wild-type S1 | 107.56 |
|  | Alpha S1 | 3041.34 |  | Alpha S1 | 59.46 |
|  | Beta S1 | 4064.57 |  | Beta S1 | 40.23 |
|  | Delta S1 | 3043.15 |  | Delta S1 | 75.75 |
|  | Omicron S1 | 2779.11 |  | Omicron S1 | 34.04 |
| W3 | Wild-type S1 | 32432.02 | C3 | Wild-type S1 | 92.03 |
|  | Alpha S1 | 9592.70 |  | Alpha S1 | 45.22 |
|  | Beta S1 | 25295.69 |  | Beta S1 | 16.84 |
|  | Delta S1 | 17134.65 |  | Delta S1 | 144.50 |
|  | Omicron S1 | 9703.74 |  | Omicron S1 | 37.17 |
| W4 | Wild-type S1 | 3126.78 | C4 | Wild-type S1 | 217.12 |
|  | Alpha S1 | 1787.77 |  | Alpha S1 | 90.63 |
|  | Beta S1 | 2551.54 |  | Beta S1 | 81.84 |
|  | Delta S1 | 2545.23 |  | Delta S1 | 118.83 |
|  | Omicron S1 | 2224.50 |  | Omicron S1 | 111.52 |
| W5 | Wild-type S1 | 940.08 | C5 | Wild-type S1 | 25.56 |
|  | Alpha S1 | 2466.29 |  | Alpha S1 | 68.40 |
|  | Beta S1 | 805.53 |  | Beta S1 | 45.84 |
|  | Delta S1 | 317.67 |  | Delta S1 | 13.33 |
|  | Omicron S1 | 529.85 |  | Omicron S1 | 96.50 |
| Neg-ctrl | Wild-type S1 | 5.93 | C6 | Wild-type S1 | 148.13 |
|  | Alpha S1 | 6.67 |  | Alpha S1 | 28.59 |
|  | Beta S1 | 9.90 |  | Beta S1 | 108.25 |
|  | Delta S1 | 13.14 |  | Delta S1 | 102.64 |
|  | Omicron S1 | 12.66 |  | Omicron S1 | 97.30 |
| Pos-ctrl | Wild-type S1 | 37069.07 | C7 | Wild-type S1 | 155.80 |
|  | Alpha S1 | 5170.12 |  | Alpha S1 | 53.13 |
|  | Beta S1 | 24684.86 |  | Beta S1 | 109.16 |
|  | Delta S1 | 11394.50 |  | Delta S1 | 84.82 |
|  | Omicron S1 | 6305.65 |  | Omicron S1 | 62.36 |

**Table S2a** Dataset of SARS-CoV-2 IgG and IgM level in sera of recovered-individuals who infected with ancestral virus (Wild-type)

|  | Sample ID | Antigen | IgM (MFI) | IgG (MFI) |
| --- | --- | --- | --- | --- |
| Wild-type recovered-individual sera | W6 | Wild-type S1 | 10.65 | 8812.28 |
|  | W7 | Wild-type S1 | 165.58 | 11128.40 |
|  | W8 | Wild-type S1 | 157.29 | 5666.80 |
|  | W9 | Wild-type S1 | 82.39 | 6415.19 |
|  | W10 | Wild-type S1 | 56.82 | 10601.12 |
|  | W11 | Wild-type S1 | 92.82 | 4622.78 |
|  | W12 | Wild-type S1 | 121.60 | 1581.08 |
|  | W13 | Wild-type S1 | 109.36 | 4301.85 |
|  | W14 | Wild-type S1 | 67.83 | 8909.88 |
|  | W15 | Wild-type S1 | 66.82 | 7221.15 |
|  | W16 | Wild-type S1 | 71.88 | 1806.81 |
|  | W17 | Wild-type S1 | 64.25 | 3258.78 |
|  | W18 | Wild-type S1 | 80.15 | 1022.71 |
|  | W19 | Wild-type S1 | 71.38 | 1170.71 |
|  | W20 | Wild-type S1 | 71.79 | 3587.55 |
|  | W21 | Wild-type S1 | 64.61 | 8106.25 |
|  | W22 | Wild-type S1 | 70.24 | 1913.67 |
|  | W23 | Wild-type S1 | 60.61 | 1404.84 |
|  | W24 | Wild-type S1 | 69.91 | 6143.65 |
|  | W25 | Wild-type S1 | 60.34 | 4780.14 |
|  | W26 | Wild-type S1 | 77.56 | 1113.40 |
|  | W27 | Wild-type S1 | 69.75 | 979.29 |
|  | W28 | Wild-type S1 | 63.82 | 2771.04 |
|  | W29 | Wild-type S1 | 63.51 | 1888.92 |
|  | W30 | Wild-type S1 | 59.57 | 1098.84 |
|  | W31 | Wild-type S1 | 46.55 | 4136.60 |
| Healthy control-individual sera | C8 | Wild-type S1 | 74.70 | 14.40 |
|  | C9 | Wild-type S1 | 22.84 | 43.26 |
|  | C10 | Wild-type S1 | 146.25 | 60.24 |
|  | C11 | Wild-type S1 | 261.81 | 9.52 |
|  | C12 | Wild-type S1 | 162.84 | 67.27 |
|  | C13 | Wild-type S1 | 246.44 | 89.70 |
|  | C14 | Wild-type S1 | 177.78 | 77.31 |
|  | C15 | Wild-type S1 | 241.02 | 61.01 |
|  | C16 | Wild-type S1 | 153.53 | 69.94 |
|  | C17 | Wild-type S1 | 203.70 | 37.56 |
|  | C18 | Wild-type S1 | 119.66 | 99.80 |
|  | C19 | Wild-type S1 | 221.65 | 39.78 |
|  | C20 | Wild-type S1 | 135.30 | 257.84 |
|  | C21 | Wild-type S1 | 231.55 | 59.78 |
|  | C22 | Wild-type S1 | 87.17 | 249.53 |
|  | C23 | Wild-type S1 | 55.60 | 35.09 |
|  | C24 | Wild-type S1 | 27.14 | 235.97 |
|  | C25 | Wild-type S1 | 32.75 | 245.52 |
|  | C26 | Wild-type S1 | 42.58 | 40.29 |
|  | C27 | Wild-type S1 | 33.49 | 58.86 |
|  | C28 | Wild-type S1 | 39.23 | 24.84 |
|  | C29 | Wild-type S1 | 14.06 | 47.80 |
|  | C30 | Wild-type S1 | 32.22 | 211.65 |

**Table S2b** Dataset of SARS-CoV-2 IgG and IgM level in sera of recovered-individuals who infected with Delta variant

|  | Sample ID | Antigen | IgM (MFI) | IgG (MFI) |
| --- | --- | --- | --- | --- |
| Delta recovered-individual sera | D1 | Delta S1 | 131.03 | 6764.20 |
|  | D2 | Delta S1 | 365.99 | 6220.48 |
|  | D3 | Delta S1 | 189.00 | 7947.13 |
|  | D4 | Delta S1 | 278.92 | 4374.90 |
|  | D5 | Delta S1 | 252.93 | 7815.91 |
|  | D6 | Delta S1 | 231.17 | 4019.10 |
|  | D7 | Delta S1 | 202.70 | 3291.81 |
|  | D8 | Delta S1 | 240.74 | 8178.22 |
|  | D9 | Delta S1 | 131.20 | 5313.09 |
|  | D10 | Delta S1 | 258.15 | 3852.47 |
|  | D11 | Delta S1 | 136.23 | 3992.49 |
|  | D12 | Delta S1 | 121.69 | 4080.28 |
|  | D13 | Delta S1 | 53.36 | 2966.03 |
|  | D14 | Delta S1 | 52.47 | 2978.38 |
| Healthy control-individual sera | C8 | Delta S1 | 4.10 | 68.29 |
|  | C9 | Delta S1 | 27.53 | 34.56 |
|  | C10 | Delta S1 | 26.26 | 14.91 |
|  | C11 | Delta S1 | 51.20 | 14.61 |
|  | C12 | Delta S1 | 21.18 | 29.12 |
|  | C13 | Delta S1 | 37.55 | 38.52 |
|  | C14 | Delta S1 | 20.19 | 5.74 |
|  | C15 | Delta S1 | 7.06 | 53.27 |
|  | C16 | Delta S1 | 60.56 | 70.05 |
|  | C17 | Delta S1 | 22.02 | 13.56 |
|  | C18 | Delta S1 | 23.44 | 44.33 |
|  | C19 | Delta S1 | 25.94 | 7.84 |
|  | C20 | Delta S1 | 20.64 | 172.05 |
|  | C21 | Delta S1 | 44.73 | 29.74 |
|  | C22 | Delta S1 | 6.68 | 41.58 |
|  | C23 | Delta S1 | 20.66 | 14.57 |
|  | C24 | Delta S1 | 5.05 | 25.61 |
|  | C25 | Delta S1 | 21.67 | 6.60 |
|  | C26 | Delta S1 | 16.45 | 5.16 |
|  | C27 | Delta S1 | 3.26 | 5.10 |
|  | C28 | Delta S1 | 15.56 | 6.34 |
|  | C29 | Delta S1 | 1.97 | 7.52 |
|  | C30 | Delta S1 | 4.58 | 24.66 |

**Table S2c** Dataset of SARS-CoV-2 IgG and IgM level in sera of recovered-individuals who infected with Omicron variant

|  | Sample ID | Antigen | IgM (MFI) | IgG (MFI) |
| --- | --- | --- | --- | --- |
| Omicron recovered-individual sera | D1 | Omicron S1 | 35.87 | 2384.66 |
|  | D2 | Omicron S1 | 124.34 | 2144.36 |
|  | D3 | Omicron S1 | 133.31 | 3083.00 |
|  | D4 | Omicron S1 | 160.73 | 2980.83 |
|  | D5 | Omicron S1 | 125.17 | 3699.08 |
|  | D6 | Omicron S1 | 153.46 | 3192.30 |
|  | D7 | Omicron S1 | 132.67 | 6349.45 |
|  | D8 | Omicron S1 | 85.81 | 3246.36 |
|  | D9 | Omicron S1 | 207.69 | 5433.83 |
|  | D10 | Omicron S1 | 97.79 | 4920.31 |
|  | D11 | Omicron S1 | 66.23 | 5379.63 |
|  | D12 | Omicron S1 | 133.76 | 5028.55 |
|  | D13 | Omicron S1 | 47.25 | 5972.78 |
|  | D14 | Omicron S1 | 16.75 | 5522.93 |
| Healthy control-individual sera | C8 | Omicron S1 | 46.10 | 13.95 |
|  | C9 | Omicron S1 | 19.30 | 8.77 |
|  | C10 | Omicron S1 | 10.39 | 9.94 |
|  | C11 | Omicron S1 | 49.23 | 5.66 |
|  | C12 | Omicron S1 | 24.51 | 9.18 |
|  | C13 | Omicron S1 | 28.69 | 3.23 |
|  | C14 | Omicron S1 | 20.00 | 15.57 |
|  | C15 | Omicron S1 | 1.78 | 9.79 |
|  | C16 | Omicron S1 | 67.99 | 12.62 |
|  | C17 | Omicron S1 | 35.60 | 9.50 |
|  | C18 | Omicron S1 | 11.36 | 12.29 |
|  | C19 | Omicron S1 | 1.58 | 5.19 |
|  | C20 | Omicron S1 | 33.48 | 7.01 |
|  | C21 | Omicron S1 | 43.67 | 19.43 |
|  | C22 | Omicron S1 | 25.12 | 9.68 |
|  | C23 | Omicron S1 | 16.74 | 6.90 |
|  | C24 | Omicron S1 | 22.52 | 16.07 |
|  | C25 | Omicron S1 | 3.24 | 21.20 |
|  | C26 | Omicron S1 | 5.47 | 11.24 |
|  | C27 | Omicron S1 | 15.33 | 5.71 |
|  | C28 | Omicron S1 | 8.77 | 12.33 |
|  | C29 | Omicron S1 | 18.25 | 25.26 |
|  | C30 | Omicron S1 | 7.95 | 7.38 |

**Table S3a** Dataset of IgG MFI of Wild-type recovered-individual sera against variants of concern

|  |  | Antigen | | |
| --- | --- | --- | --- | --- |
|  |  | Wild-type S1 | Delta S1 | Omicron S1 |
|  | Sample ID | MFI | | |
| Wild-type recovered-individual sera | W6 | 8812.28 | 6814.91 | 3456.68 |
|  | W7 | 11128.40 | 7047.56 | 1271.49 |
|  | W8 | 5666.80 | 4491.80 | 3180.44 |
|  | W9 | 6415.19 | 6747.54 | 178.58 |
|  | W10 | 10601.12 | 7442.02 | 349.91 |
|  | W11 | 4622.78 | 3598.32 | 573.18 |
|  | W12 | 1581.08 | 2882.17 | 291.80 |
|  | W13 | 4301.85 | 3463.49 | 1537.64 |
|  | W14 | 8909.88 | 8311.27 | 1476.05 |
|  | W15 | 7221.15 | 4857.54 | 1876.30 |
|  | W16 | 1806.81 | 2243.25 | 84.99 |
|  | W17 | 3258.78 | 1793.11 | 744.89 |
|  | W18 | 1022.71 | 4756.55 | 342.96 |
|  | W19 | 1170.71 | 1281.41 | 390.37 |
|  | W20 | 3587.55 | 1479.55 | 1776.06 |
|  | W21 | 8106.25 | 6764.39 | 386.02 |
|  | W22 | 1913.67 | 6249.62 | 913.72 |
|  | W23 | 1404.84 | 2959.57 | 601.89 |
|  | W24 | 6143.65 | 4872.18 | 148.81 |
|  | W25 | 4780.14 | 6709.89 | 93.48 |
|  | W26 | 1113.40 | 5451.52 | 223.73 |
|  | W27 | 979.29 | 952.07 | 1093.56 |
|  | W28 | 2771.04 | 3166.78 | 144.38 |
|  | W29 | 1888.92 | 1215.21 | 895.88 |
|  | W30 | 1098.84 | 753.15 | 97.31 |
|  | W31 | 4136.60 | 1187.32 | 354.37 |

**Table S3b** Dataset of IgG MFI of Delta recovered-individual sera against variants of concern

|  |  | Antigen | | |
| --- | --- | --- | --- | --- |
|  |  | Wild-type S1 | Delta S1 | Omicron S1 |
|  | Sample ID | MFI | | |
| Delta recovered-individual sera | D1 | 2821.69 | 6764.20 | 740.21 |
|  | D2 | 3446.26 | 6220.48 | 941.24 |
|  | D3 | 4134.42 | 7947.13 | 1165.99 |
|  | D4 | 4361.11 | 4374.90 | 960.20 |
|  | D5 | 7510.19 | 7815.91 | 909.46 |
|  | D6 | 7304.55 | 4019.10 | 525.35 |
|  | D7 | 2952.03 | 3291.81 | 456.83 |
|  | D8 | 4375.78 | 8178.22 | 523.89 |
|  | D9 | 3267.62 | 5313.09 | 388.47 |
|  | D10 | 3595.18 | 3852.47 | 372.61 |
|  | D11 | 3311.19 | 3992.49 | 416.85 |
|  | D12 | 3270.83 | 4080.28 | 381.84 |
|  | D13 | 3117.25 | 2966.03 | 528.84 |
|  | D14 | 3290.81 | 2978.38 | 467.44 |

**Table S3c** Dataset of IgG MFI of Omicron recovered-individual sera against variants of concern

|  |  | Antigen | | |
| --- | --- | --- | --- | --- |
|  |  | Wild-type S1 | Delta S1 | Omicron S1 |
|  | Sample ID | MFI | | |
| Omicron recovered-individual sera | O1 | 472.59 | 99.82 | 2384.66 |
|  | O2 | 650.51 | 147.56 | 2144.36 |
|  | O3 | 338.97 | 61.16 | 3083.00 |
|  | O4 | 1202.32 | 256.79 | 2980.83 |
|  | O5 | 592.53 | 57.17 | 3699.08 |
|  | O6 | 179.36 | 103.49 | 3192.30 |
|  | O7 | 895.73 | 25.37 | 6349.45 |
|  | O8 | 578.52 | 87.76 | 3246.36 |
|  | O9 | 4696.06 | 617.66 | 5433.83 |
|  | O10 | 1871.63 | 217.72 | 4920.31 |
|  | O11 | 2536.41 | 179.44 | 5379.63 |
|  | O12 | 687.80 | 28.06 | 5028.55 |
|  | O13 | 1677.93 | 205.34 | 5972.78 |
|  | O14 | 575.23 | 59.26 | 5522.93 |

**Table S4** Dataset of IgG level and avidity of a volunteer who injected COVID-19 vaccine at different time

|  |  |  | Urea | No urea |  |
| --- | --- | --- | --- | --- | --- |
| Sample ID | Antigen | Date of injection | MFI | MFI | Avidity |
| V1 | Wild-type S1 | June 27 (the day before the second dose) | 22.27 | 446.45 | 0.05 |
|  |  | July 4 (one week after the second dose) | 913.89 | 4179.35 | 0.22 |
|  |  | August 28 (seven weeks after the second dose) | 1486.08 | 5773.29 | 0.26 |
|  | Alpha S1 | June 27 (the day before the second dose) | 14.20 | 298.80 | 0.05 |
|  |  | July 4 (one week after the second dose) | 713.05 | 3164.76 | 0.23 |
|  |  | August 28 (seven weeks after the second dose) | 913.78 | 3825.53 | 0.24 |
|  | Beta S1 | June 27 (the day before the second dose) | 7.79 | 172.77 | 0.05 |
|  |  | July 4 (one week after the second dose) | 304.03 | 2757.98 | 0.11 |
|  |  | August 28 (seven weeks after the second dose) | 522.55 | 4142.08 | 0.13 |
|  | Delta S1 | June 27 (the day before the second dose) | 5.73 | 129.63 | 0.04 |
|  |  | July 4 (one week after the second dose) | 136.37 | 1223.22 | 0.11 |
|  |  | August 28 (seven weeks after the second dose) | 260.66 | 1935.59 | 0.13 |
|  | Omicron S1 | June 27 (the day before the second dose) | 9.44 | 119.53 | 0.08 |
|  |  | July 4 (one week after the second dose) | 92.65 | 617.69 | 0.15 |
|  |  | August 28 (seven weeks after the second dose) | 180.01 | 1055.32 | 0.17 |

**Table S5a** Dataset of IgG level and avidity of individuals who recovered from Wild-type infection

|  |  |  |  | Urea | No urea |
| --- | --- | --- | --- | --- | --- |
| Sample ID | Days since symptom onset to collect sera | Antigen | MFI | MFI | Avidity |
| W32 | 196 | Wild-type S1 | 32853.76 | 40597.08 | 0.81 |
|  |  | Alpha S1 | 7388.43 | 17036.83 | 0.43 |
|  |  | Beta S1 | 15646.58 | 39433.57 | 0.40 |
|  |  | Delta S1 | 14282.26 | 24148.14 | 0.59 |
|  |  | Omicron S1 | 7771.83 | 13825.19 | 0.56 |
| W33 | 197 | Wild-type S1 | 13742.79 | 27091.54 | 0.51 |
|  |  | Alpha S1 | 3385.31 | 9835.68 | 0.34 |
|  |  | Beta S1 | 5147.47 | 22026.10 | 0.23 |
|  |  | Delta S1 | 3042.11 | 9873.45 | 0.31 |
|  |  | Omicron S1 | 4708.70 | 6737.90 | 0.70 |
| W34 | 197 | Wild-type S1 | 2735.91 | 3967.09 | 0.69 |
|  |  | Alpha S1 | 20.35 | 798.21 | 0.03 |
|  |  | Beta S1 | 132.66 | 1057.97 | 0.13 |
|  |  | Delta S1 | 12.28 | 466.04 | 0.03 |
|  |  | Omicron S1 | 440.70 | 582.11 | 0.76 |
| W35 | 204 | Wild-type S1 | 3724.66 | 13042.43 | 0.29 |
|  |  | Alpha S1 | 409.45 | 7234.77 | 0.06 |
|  |  | Beta S1 | 542.08 | 4269.34 | 0.13 |
|  |  | Delta S1 | 154.08 | 1555.00 | 0.10 |
|  |  | Omicron S1 | 5583.06 | 14670.98 | 0.38 |
| W36 | 206 | Wild-type S1 | 7851.18 | 11919.24 | 0.66 |
|  |  | Alpha S1 | 2452.10 | 2516.46 | 0.97 |
|  |  | Beta S1 | 9524.69 | 11403.45 | 0.84 |
|  |  | Delta S1 | 4081.46 | 5075.88 | 0.80 |
|  |  | Omicron S1 | 93.53 | 476.72 | 0.20 |
| W37 | 339 | Wild-type S1 | 8893.34 | 27470.70 | 0.32 |
|  |  | Alpha S1 | 3018.27 | 3211.63 | 0.94 |
|  |  | Beta S1 | 6550.92 | 7481.60 | 0.88 |
|  |  | Delta S1 | 5293.88 | 8287.72 | 0.64 |
|  |  | Omicron S1 | 715.86 | 3471.45 | 0.21 |
| W38 | 341 | Wild-type S1 | 5719.37 | 6603.11 | 0.87 |
|  |  | Alpha S1 | 98.24 | 1230.31 | 0.08 |
|  |  | Beta S1 | 484.39 | 1596.52 | 0.30 |
|  |  | Delta S1 | 98.22 | 926.48 | 0.11 |
|  |  | Omicron S1 | 2389.44 | 6667.82 | 0.36 |
| W39 | 355 | Wild-type S1 | 16065.27 | 24728.99 | 0.65 |
|  |  | Alpha S1 | 3117.53 | 7118.15 | 0.44 |
|  |  | Beta S1 | 5201.72 | 15296.14 | 0.34 |
|  |  | Delta S1 | 2849.70 | 8197.70 | 0.35 |
|  |  | Omicron S1 | 4054.98 | 4895.68 | 0.83 |
| W40 | 355 | Wild-type S1 | 6198.89 | 12963.32 | 0.48 |
|  |  | Alpha S1 | 1020.74 | 3165.12 | 0.32 |
|  |  | Beta S1 | 1622.39 | 3558.40 | 0.46 |
|  |  | Delta S1 | 1012.27 | 2369.59 | 0.43 |
|  |  | Omicron S1 | 11318.70 | 14795.77 | 0.76 |
| W41 | 357 | Wild-type S1 | 12727.40 | 34431.43 | 0.37 |
|  |  | Alpha S1 | 2576.53 | 8071.06 | 0.32 |
|  |  | Beta S1 | 6749.78 | 18844.51 | 0.36 |
|  |  | Delta S1 | 4133.71 | 9340.14 | 0.44 |
|  |  | Omicron S1 | 5488.03 | 27223.61 | 0.20 |
| W42 | 491 | Wild-type S1 | 3896.47 | 5231.17 | 0.74 |
|  |  | Alpha S1 | 470.84 | 482.25 | 0.98 |
|  |  | Beta S1 | 1120.05 | 2455.21 | 0.46 |
|  |  | Delta S1 | 1589.53 | 2304.38 | 0.69 |
|  |  | Omicron S1 | 1907.19 | 2022.72 | 0.94 |
| W43 | 512 | Wild-type S1 | 14358.19 | 14834.89 | 0.97 |
|  |  | Alpha S1 | 4355.40 | 5332.44 | 0.82 |
|  |  | Beta S1 | 4913.88 | 5954.46 | 0.83 |
|  |  | Delta S1 | 1875.92 | 3619.02 | 0.52 |
|  |  | Omicron S1 | 1989.70 | 2374.15 | 0.84 |
| W44 | 513 | Wild-type S1 | 10125.21 | 10486.10 | 0.97 |
|  |  | Alpha S1 | 7456.87 | 8713.05 | 0.86 |
|  |  | Beta S1 | 15947.31 | 21782.33 | 0.73 |
|  |  | Delta S1 | 8071.70 | 9308.33 | 0.87 |
|  |  | Omicron S1 | 5462.09 | 5658.57 | 0.97 |
| W45 | 516 | Wild-type S1 | 6323.75 | 8341.98 | 0.76 |
|  |  | Alpha S1 | 1379.90 | 1930.06 | 0.71 |
|  |  | Beta S1 | 3861.71 | 4902.30 | 0.79 |
|  |  | Delta S1 | 1587.39 | 1845.90 | 0.86 |
|  |  | Omicron S1 | 3182.26 | 4007.73 | 0.79 |
| W46 | 530 | Wild-type S1 | 5264.57 | 7903.81 | 0.67 |
|  |  | Alpha S1 | 931.77 | 1308.85 | 0.71 |
|  |  | Beta S1 | 4453.73 | 4864.76 | 0.92 |
|  |  | Delta S1 | 2352.29 | 3279.64 | 0.72 |
|  |  | Omicron S1 | 5376.21 | 7804.38 | 0.69 |

**Table S5b** Dataset of IgG level and avidity of volunteers who completed two doses of COVID-19 vaccine

|  |  |  | Urea | No urea |  |
| --- | --- | --- | --- | --- | --- |
| Sample ID | Days since completed vaccination to collect sera | Antigen | MFI | MFI | Avidity |
| V2 | 27 | Wild-type S1 | 2081.20 | 7527.90 | 0.28 |
|  |  | Alpha S1 | 670.00 | 2019.11 | 0.33 |
|  |  | Beta S1 | 246.96 | 409.79 | 0.60 |
|  |  | Delta S1 | 528.22 | 1666.38 | 0.32 |
|  |  | Omicron S1 | 85.68 | 221.32 | 0.39 |
| V3 | 31 | Wild-type S1 | 578.01 | 4179.28 | 0.14 |
|  |  | Alpha S1 | 241.36 | 1250.95 | 0.19 |
|  |  | Beta S1 | 58.66 | 333.93 | 0.18 |
|  |  | Delta S1 | 2326.41 | 2647.38 | 0.88 |
|  |  | Omicron S1 | 60.86 | 268.09 | 0.23 |
| V4 | 32 | Wild-type S1 | 2826.06 | 19900.57 | 0.14 |
|  |  | Alpha S1 | 174.11 | 745.31 | 0.23 |
|  |  | Beta S1 | 97.11 | 209.89 | 0.46 |
|  |  | Delta S1 | 1139.03 | 4649.95 | 0.24 |
|  |  | Omicron S1 | 500.08 | 1704.51 | 0.29 |
| V5 | 36 | Wild-type S1 | 311.79 | 2623.59 | 0.12 |
|  |  | Alpha S1 | 273.13 | 850.51 | 0.32 |
|  |  | Beta S1 | 72.40 | 178.11 | 0.41 |
|  |  | Delta S1 | 310.47 | 1039.25 | 0.30 |
|  |  | Omicron S1 | 35.21 | 142.45 | 0.25 |
| V6 | 39 | Wild-type S1 | 73.27 | 2073.68 | 0.04 |
|  |  | Alpha S1 | 206.53 | 1248.89 | 0.17 |
|  |  | Beta S1 | 53.34 | 277.38 | 0.19 |
|  |  | Delta S1 | 84.98 | 385.80 | 0.22 |
|  |  | Omicron S1 | 1.53 | 7.63 | 0.20 |
| V7 | 42 | Wild-type S1 | 2034.61 | 30545.05 | 0.07 |
|  |  | Alpha S1 | 636.50 | 3025.23 | 0.21 |
|  |  | Beta S1 | 342.65 | 1278.33 | 0.27 |
|  |  | Delta S1 | 4066.82 | 15867.77 | 0.26 |
|  |  | Omicron S1 | 360.94 | 1585.78 | 0.23 |
| V8 | 49 | Wild-type S1 | 3169.37 | 20175.10 | 0.16 |
|  |  | Alpha S1 | 1567.83 | 6956.42 | 0.23 |
|  |  | Beta S1 | 1448.51 | 3210.24 | 0.45 |
|  |  | Delta S1 | 4053.77 | 8847.20 | 0.46 |
|  |  | Omicron S1 | 763.91 | 1510.71 | 0.51 |
| V9 | 49 | Wild-type S1 | 10020.10 | 45986.77 | 0.22 |
|  |  | Alpha S1 | 2650.56 | 4122.38 | 0.64 |
|  |  | Beta S1 | 1030.08 | 1441.77 | 0.71 |
|  |  | Delta S1 | 6128.51 | 16851.72 | 0.36 |
|  |  | Omicron S1 | 1687.25 | 3356.99 | 0.50 |
| V10 | 50 | Wild-type S1 | 6962.70 | 28287.09 | 0.25 |
|  |  | Alpha S1 | 844.90 | 2150.81 | 0.39 |
|  |  | Beta S1 | 585.28 | 1014.05 | 0.58 |
|  |  | Delta S1 | 3815.04 | 13677.91 | 0.28 |
|  |  | Omicron S1 | 1098.06 | 2578.13 | 0.43 |
| V11 | 50 | Wild-type S1 | 1543.50 | 5006.51 | 0.31 |
|  |  | Alpha S1 | 838.64 | 1359.18 | 0.62 |
|  |  | Beta S1 | 335.28 | 600.94 | 0.56 |
|  |  | Delta S1 | 1718.51 | 2210.75 | 0.78 |
|  |  | Omicron S1 | 219.27 | 253.22 | 0.87 |
| V12 | 51 | Wild-type S1 | 573.21 | 5200.10 | 0.11 |
|  |  | Alpha S1 | 243.35 | 1336.60 | 0.18 |
|  |  | Beta S1 | 210.73 | 567.28 | 0.37 |
|  |  | Delta S1 | 627.68 | 1925.20 | 0.33 |
|  |  | Omicron S1 | 56.66 | 108.26 | 0.52 |
| V13 | 51 | Wild-type S1 | 980.33 | 5706.23 | 0.17 |
|  |  | Alpha S1 | 207.81 | 1138.71 | 0.18 |
|  |  | Beta S1 | 177.78 | 817.54 | 0.22 |
|  |  | Delta S1 | 660.71 | 3185.62 | 0.21 |
|  |  | Omicron S1 | 161.30 | 603.89 | 0.27 |
| V14 | 51 | Wild-type S1 | 352.99 | 4197.61 | 0.08 |
|  |  | Alpha S1 | 322.34 | 1517.38 | 0.21 |
|  |  | Beta S1 | 219.28 | 643.35 | 0.34 |
|  |  | Delta S1 | 383.50 | 2111.96 | 0.18 |
|  |  | Omicron S1 | 75.78 | 286.59 | 0.26 |
